# Innovation in sweet rice wine with high antioxidant activity: *Eucommia ulmoides* leaf sweet rice wine

**DOI:** 10.3389/fnut.2022.1108843

**Published:** 2023-01-10

**Authors:** Na Ren, Weiwei Gong, Yichen Zhao, De-gang Zhao, Yiwen Xu

**Affiliations:** ^1^Key Laboratory of Plant Resources Conservation and Germplasm Innovation in the Mountainous Region (Ministry of Education), College of Tea Sciences, Guizhou University, Guiyang, China; ^2^Plant Conservation Technology Center, Guizhou Key Laboratory of Agricultural Biotechnology, Guizhou Academy of Agricultural Sciences, Guiyang, China; ^3^Guizhou Sauce Wine Group Liquor Production Co., Ltd., Guiyang, China

**Keywords:** *Eucommia ulmoides* leaves, sweet rice wine, flavonoids, metabolome, antioxidant activity, antihyperglycemic, antihyperlipidemic, antihypertensive

## Abstract

The dried leaves of *Eucommia ulmoides* Oliv., which have a high nutritional value, are mainly used in both medicine and food. In this study, we used *Eucommia ulmoides* leaf superfine powder as an additive in the fermentation of glutinous rice (*Semen Oryzae Glutinosae*) to develop a new healthcare product, Eucommia leaf sweet rice wine. The fermentation conditions were optimized, and the nutrient value was evaluated through analyses of metabolites, functional compositions, antioxidant capacity, and antihyperglycemic, antihyperlipidemic, and antihypertensive abilities. The metabolic analysis demonstrated that Eucommia leaf sweet rice wine contained a large number of flavonoids and other metabolites. Eucommia leaf sweet rice wine had higher contents of flavonoid (729.0 ± 0.11 μg/g), free amino acids (55.0 ± 0.37 μg/g), polyphenol (150.0 ± 0.43 μg/g), and polysaccharide (0.25 ± 0.03 μg/g) than traditional sweet rice wine, with increases of 14.7, 2.6, 6.8, and 6.3 times, respectively. In addition, an analysis of antioxidant capacity *in vitro* revealed that Eucommia leaf sweet rice wine had a high level of activity in scavenging 2, 2-diphenyl-1-picrylhydrazyl (DPPH), superoxide anion, and hydroxyl radicals, as well as in reducing iron, indicating that it was a strong antioxidant. Furthermore, Eucommia leaf sweet rice wine had a high cholate binding capacity and could significantly inhibit α-amylase, α-glucosidase, and angiotensin-converting enzyme (ACE) activity. In conclusion, this study developed a new application of Eucommia leaf in sweet rice wine fermentation and brewed Eucommia leaf sweet rice wine with strong antioxidant activity and positive antihypertensive, antihyperglycemic, and antihyperlipidemic effects *in vitro*. This study suggests new opportunities for the wider use of *Eucommia ulmoides* leaves and adds variety to sweet rice wine.

## 1. Introduction

*Eucommia ulmoides* Oliv. is a plant of the family Eucommiaceae, which belongs to only one genus and is a perennial dioecious deciduous tree (1). *Eucommia ulmoides* leaf is a dried leaf of *Eucommia ulmoides* Oliv. (2). *Eucommia ulmoides* leaves are used in both medicine and food and are known for their health benefits and high nutrient content (3). Polyphenols and flavonoids are some of the main effective components of *Eucommia ulmoides* leaves (4, 5). They are good functional food additives and have good antitussive, expectorant, liver protection, antioxidation, and antiaging effects (6, 7). Flavonoids are a type of plant pigment that has a variety of health benefits. Since the human body cannot produce these compounds, it is important to get them from plant sources. Jia et al. extracted flavonoids from *Eucommia ulmoides* leaves and proved that the flavonoids in *Eucommia ulmoides* leaves had a stronger scavenging effect on hydroxyl free radicals and superoxide anions (8). Therefore, *Eucommia ulmoides* leaves have strong antioxidant activity due to the presence of flavonoids. In pharmacological studies, *Eucommia ulmoides* leaves contain cyclopropanoids, lignans, phenylpropanoids, flavonoids, and ketocompounds; thus, they have a variety of health benefits, such as hypoglycemic, hypotensive, hypolipidemic, anti-inflammatory, neuroprotective, and antiaging activities (9–11).

Sweet fermented rice wine, a traditional food in China, is made from steamed glutinous rice (*Semen Oryzae Glutinosae*) and fermented with sweet koji (12). The low-sugar contents of sweet fermented rice wine are easily absorbed by the human body and can quickly replenish energy and strengthen the stomach and the spleen (13). There are currently numerous healthcare products on the market that combine Chinese medicinal ingredients with sweet rice wine used for both medicinal and culinary purposes. Fu et al. used Taizi ginseng and glutinous rice as the main raw materials, and the brewed Taizi ginseng and glutinous rice sweet wine have a unique taste and rich nutritional value (14). It has the advantage of enhancing immune function, acting as an anti-fatigue agent, and increasing appetite (15, 16). Li et al. developed *Pueraria lobata* glutinous rice wine, which protects myocardial cells and improves memory and antioxidation (17). These studies suggest that the development of sweet rice wine that is good for health has greatly improved the nutritional value of traditional sweet rice wine.

In recent years, *Eucommia ulmoides* bark and *Eucommia ulmoides* gum have been widely developed and applied; however, the utilization rate of *Eucommia ulmoides* leaf is alarmingly low (18). *Eucommia ulmoides* leaves have been used in gruel, tea, and beverages (19, 20). However, the production of *Eucommia ulmoides* leaf health products is conducted in a relatively blind manner, the quality of the products is poor, and the variety of the products is inadequate (21). In addition, people have increasingly paid attention to the use of non-natural antioxidant products such as drugs and food additives. As a natural plant with multiple functions, developing products from *Eucommia ulmoides* leaves is significant. Based on this fact, in this study, *Eucommia ulmoides* leaf superfine powder and glutinous rice were used as raw materials for fermentation, the fermentation processes were optimized, and the nutritional value was evaluated to obtain a functional food with a unique flavor. We believe that this study can not only improve the nutritional value of sweet fermented rice wine but also show that *Eucommia ulmoides* leaves can be effectively utilized. In addition, we wish to provide a new perspective on the healthy development of the *Eucommia ulmoides* industry.

## 2. Materials and methods

### 2.1. Materials

Fresh leaves of *Eucommia ulmoides* Oliv. were harvested in June, and the *Eucommia ulmoides* leaf superfine powder (particle diameter less than 50 μm) was provided by Anshun Yucha Village Tea Industry Co., Ltd. (Guizhou, China). Wuchang short glutinous rice (*japonica* rice) was purchased from Wuchang Deqiang Rice Industry Co., Ltd. (Harbin, China). Angel Sweet Liquor was obtained from Hubei Angel Yeast Co., Ltd. (Hubei, China).

#### 2.1.1. *Eucommia ulmoides* leaf superfine powder processing

The fresh leaves of *Eucommia ulmoides* were collected, the stems and leaves were separated after being steamed at 550°C, the leaves were dried at 85°C, and the metal and foreign matter were detected prior to packaging.

#### 2.1.2. Eucommia leaf sweet rice wine processing

First, an appropriate amount of glutinous rice was weighed and rinsed in a clean container until there was no foreign matter. Next, we filled a container with clean water two times the amount of glutinous rice and allowed it to soak for approximately 12 h at room temperature until it could be crushed by hand. Then, we drained the soaked glutinous rice and steamed it in a steamer for 30 min. Second, the glutinous rice was dispersed in pure water and allowed to cool to approximately 35°C, after which a certain proportion of Eucommia leaf superfine powder and koji was added to the glutinous rice and stirred well. We flattened the glutinous rice balls in a fermenter, then poked a hollow in the middle of the container with a stick and filled it with a certain proportion of pure water. The sealed fermenter was fermented at a certain temperature in a constant-temperature incubator, and the fermentation product was subsequently obtained. Finally, the product was placed in a water bath at 75°C for water bath sterilization for 15 min to obtain the new product.

### 2.2. Sensory evaluation and optimization of fermentation processes

The flavor and taste of the new product were assessed through sensory evaluation. In each test, 10 sensory evaluators (five men and five women) evaluated and scored the product separately. The sensory evaluation of the glutinous rice mixed with *Eucommia ulmoides* leaves was based on four parameters: smell, taste, color, and shape (for a full score of 100 points).

Four factors, fermentation temperature, fermentation time, amount of koji, and amount of Eucommia superfine leaf powder, were selected for single-factor analysis and a subsequent L_9_(4^3^) orthogonal experiment, combined with the sensory evaluation so as to explore the optimal fermentation conditions of Eucommia leaf sweet rice wine. Details are shown in the Supplementary Tables.

### 2.3. Physical, chemical, and hygiene index testing

The total sugar content was determined in accordance with the anthrone-sulfuric acid colorimetric method, and the details are shown in the Supplementary Tables. The content of total acid (calculated as lactic acid) was determined, as reported by Wang et al. (22); the alcohol content was determined using the alcohol meter method, and the pH value was measured with a pH meter.

The lead content was determined using the procedure used by Nikpooyan et al. (23). The inorganic arsenic content was determined in accordance with GB 5009.11-2014, “Determination of total arsenic and inorganic arsenic in food.” The content of aflatoxin B1 was determined using the same method as that used by Ren et al. (24). The number of colonies of *Escherichia coli, Staphylococcus aureus*, and *Salmonella* were determined in accordance with GB/T 4789.25-2003, “Food Hygiene Microbiological Inspection Alcohol Inspection.”

### 2.4. Metabolic analysis

#### 2.4.1. Sample preparation and extraction

After thawing the samples of CK1, CK2, and CK3 (traditional sweet rice wine) and DZ1, DZ2, and DZ3 (Eucommia leaf sweet rice wine) from the refrigerator at −80°C, they were vortexed for 10 s. Then, 2 mL of the mixed sample was taken and placed in a 10 mL centrifuge tube, and the sample was immersed in liquid nitrogen as a whole. After the sample was completely frozen, it was placed in a lyophilizer for lyophilization. After the samples were completely lyophilized, 400 μL of a 70% methanol internal standard extract was added. The mixture was centrifuged (12,000 r/min, 4 °C) for 3 min. The supernatant was filtered with a microporous filter membrane (0.22 μm) and stored in a sample flask for LC-MS/MS analysis. The UPLC conditions and ESI-Q TRAP-MS/MS are shown in the Supplementary Tables.

### 2.5. Analysis of main nutrient compositions and functional compositions

The free amino acid content was determined using the same method as that used by Shao et al. (25). In addition, the polyphenol content was determined in the same manner as Luo et al. (26). The flavonoid and total polysaccharide contents of the samples were estimated using a spectrophotometer. The rutin, chlorogenic acid, and catechin (EGCG) contents of the samples were analyzed by high-performance liquid chromatography (HPLC), and all the detailed processes used are shown in the Supplementary Tables.

### 2.6. Analysis of antioxidant activity of the new product *in vitro*

#### 2.6.1. Determination of the scavenging rate of 2, 2-diphenyl-1-picrylhydrazyl

First, 1 mL of a 0.2 mmol/L DPPH anhydrous ethanol solution and 0.5 mL of sample were mixed in a colorimetric tube, shaken well with a shaker, and kept in the dark for 30 min. Second, the mixture was centrifuged at 6,000 r/min for 10 min, the supernatant was collected, and the absorbance A1 was measured at 517 nm. In the blank group, A0, we replaced the DPPH solution with absolute ethanol. In the control group (A2), the sample solution was replaced with distilled water. Three parallel trials were carried out.


(1) $$\begin{array}{l} \text{DPPH free radical scavenging rate (\%)}=\text{1-}\frac{\left(A\text{1-}A\text{0}\right)}{A\text{2}}\times 100 \end{array}$$

#### 2.6.2. Determination of the scavenging rate of superoxide anion (O2-·)

Samples (0.1, 0.2, 0.5, 0.8, and 1.0 mL) were placed into six test tubes, and distilled water was used to make the mixture up to 1 mL (the same below). Then, 4.5 mL of a 50 mmol/L Tris-HCl buffer solution (preheated for 20 min in a 25°C water bath) and 0.4 mL of a 25 mmol/L pyrogallol solution were added. The mixture was reacted in a 25°C water bath for 5 min. Then, 1 mL of an 8 mol/L HCl solution was added to terminate the reaction, and the absorbance A1 was measured at 299 nm. For the blank control, we used distilled water instead of the sample solution and measured the absorbance value of A0. We replaced the pyrogallol solution with the same volume of distilled water and measured the absorbance A2 of the sample solution in the system.


(2) $$\begin{array}{l} \text{Superoxide anion scavenging rate (\%)}=\frac{A\text{0-}\left(A1\text{-}A2\right)}{A0}\times 100 \end{array}$$

#### 2.6.3. Determination of the scavenging rate of hydroxyl radicals (·OH)

First, 2 mL of a 6 mmol/L FeSO_4_ solution and 2 mL of a 6 mmol/L H_2_O_2_ solution were added to the samples. The mixture was shaken well and allowed to stand for 10 min. Second, 2 mL of a 6 mmol/L salicylic acid solution was added, shaken well, allowed to stand for 30 min, and centrifuged at 5,000 r/min for 5 min, and the absorbance value A1 was measured at 510 nm. For the blank control, distilled water was used instead of the sample solution, and the absorbance value A0 was measured. We replaced the salicylic acid solution with the same volume of distilled water and measured the absorbance A2 of the sample solution in the system.


(3) $$\begin{array}{l} \text{Hydroxyl radical scavenging rate (\%)}=\frac{A\text{0-}\left(A1\text{-}A2\right)}{A0}\times 100 \end{array}$$

#### 2.6.4. Determination of iron reducing capacity

First, 2.5 mL of 0.2 mol/L phosphate buffer solution (pH=6.6) was added to the samples. Second, a 2.5 mL solution of 1% potassium ferricyanide was added, thoroughly mixed, and reacted in a water bath at 50°C for 20 min before being allowed to cool rapidly. Ten percent trichloroacetic acid was added to 1 mL of the sample, mixed well, and centrifuged at 3000 r/min for 10 min. Then, 2.5 mL of the supernatant was taken, 2.5 mL of distilled water, 0.5 mL of 0.1% ferric chloride were added, mixed well, and allowed to stand for 10 min, after which absorbance was measured at 700 nm. The higher the absorbance, the better the reducibility.

### 2.7. Enzyme inhibition ability *in vitro*

#### 2.7.1. Analysis of α-amylase and α-glucosidase inhibition effect

The α-glucosidase and α-amylase inhibition experiment was slightly modified, as suggested by Apostolidis et al. (27).

For the α-glucosidase inhibition experiment, 5 μL of sample solution, 25 μL of α-glucosidase (0.1 U/mL), and 140 μL of 0.1 mol/L phosphate buffer (PBS buffer, pH = 6.8) were mixed in a tube and reacted at 37°C for 5 min. Then, 25 μL of 24 mmol/L p-nitrophenyl-β-D-galactopyranoside (PNPG) solution was added and reacted at 37°C for 15 min. Finally, 100 μL of a 0.2 mol/L Na_2_CO_3_ termination solution was added, and the absorbance was measured at 405 nm. The sample solution was replaced by distilled water as a blank, and α-glucosidase was replaced by distilled water as a control. The experiment was repeated in three groups.


(4) $$\begin{array}{l} \text{Inhibition rate (\%)}=\frac{\text{1-}\left(Asample\text{-}Acontrol\right)}{Ablank}\times 100\% \end{array}$$

For the α-amylase inhibition experiment, 500 μL of sample solution was mixed with 500 μL of α-amylase solution (1 U/mL) and incubated at 25°C for 10 min. Then, 500 μL of the soluble starch solution was added and incubated at 25°C for 10 min. The reaction was terminated by adding 1 mL of DNS reagent, boiling the sample solution in a water bath for 5 min, cooling it to room temperature, and diluting it to 10 mL with distilled water. The absorbance A1 was measured at 520 nm. PBS buffer was used to replace the solution to be tested, and the absorbance A2 was obtained. The absorbances of A0 and A3 were measured after the solution, and the α-amylase solution was replaced with distilled water and PBS buffer, respectively.


(5) $$\begin{array}{l} \text{Inhibition rate (\%)}=\frac{\text{1-}\left(A1\text{-}A2\right)}{\left(A0\text{-}A3\right)}\times \text{100\%} \end{array}$$

#### 2.7.2. Cholate binding experiment

The experimental steps were recommended by Zhou et al. with some modifications (28).

Standard cholic curve: First, 2 mL of standard solutions of different concentrations were placed in different stopper test tubes, and 6 mL of an H_2_SO_4_ solution with a 60% mass fraction was added to the water bath at 70°C for 20 min, and the ice bath was removed for 5 min. Absorbance was measured at 387 nm. The standard curves of sodium taurocholate and sodium glycocholate were drawn with the content of cholate as the horizontal coordinate and absorbance as the vertical coordinate.

Then, 3 mL of the sample solution was removed from a 10 mL stopper flask, and 3 mL of a 10 mg/mL pepsin solution and 1 mL of a 0.01 mol/L HCl solution were added to each flask. The digestion oscillated in a constant temperature water bath at 37°C for 1 h. Then, the pH value was adjusted to 6.3 with a 0.1 mol/L sodium hydroxide solution before adding 4 mL of a 10 mg/mL trypsin solution, which was oscillated and digested in a constant temperature water bath at 37°C for 1 h. Each sample was further added to 4 mL of 0.4 mmol/L sodium glycocholate or 4 mL of 0.5 mmol/L sodium taurocholate, incubated in a constant temperature water bath oscillation for 1 h, and centrifuged at 4,000 r/min for 20 min. The supernatant was taken to determine the absorbance at 387 nm. The residual sodium taurocholate and sodium glycocholate contents were calculated according to the standard curves. The binding rate was expressed as a percentage of the difference between the entire amount of sodium taurocholate and sodium glycocholate added and the remaining amount.

#### 2.7.3. Angiotensin-converting enzyme inhibition activity

The ACE inhibitory activity was measured using the method recommended by Martin et al. after some modifications (29). N-(3-(2-furyl)acryloyl)-L-phenylalanyl-glycyl-glycine (FAPGG) was used as the substrate for ACE, and each reaction component was added according to Table 1. A 40 μL sample and N-2-hydroxyethyl piperazine-N-ethane-sulphonic acid (HEPES) buffer (80 mmol/L, pH = 8.3) were added to a 96-well plate and then mixed with a 10 μL 0.1 U/mL ACE solution. Finally, 50 μL of FAPGG solution was added, and the heat was held at 37°C for 5 min. The absorption value was determined at a wavelength of 340 nm. The heat was held for 30 min, and the absorption value was measured again.


(6) $$\begin{array}{l} \text{Inhibition rate (\%)}=\frac{\Delta A\text{c-}\Delta As}{\Delta As}\times 100, \end{array}$$

where ΔAc is the change in the light absorption value in the blank group within 30 min, and ΔAs is the change in the absorption value of the sample group within 30 min.

**Table 1:** Method for determination of angiotensin-converting enzyme (ACE) inhibitory activity.

**Reagent**	**Control/μL**	**Sample/μL**
ACE	10	10
FAPGG	50	50
HEPES buffer	40	0
Sample	0	40

### 2.8. Data analysis

All data were presented as the mean ± standard deviation. A *p*-value < 0.05, 0.01, or 0.001 was considered statistically significant. We used SPSS 23.0 software to perform a variance analysis on the experimental data. GraphPad Prism 8 software was used to draw line graphs and histograms, and Origin 95-64 software was used to draw high-performance liquid chromatograms. Each experiment was repeated three times. An unsupervised principal component analysis (PCA) was performed using the statistics function prcomp within R (www.r-project.org). Before performing unsupervised PCA, we scaled the data to have unit variance. For two-group analysis, differential metabolites were determined using the variable importance in projection (VIP) criteria (VIP ≥ 1) and the absolute log_2_FC criteria (|Log_2_FC| ≥ 1.0).

## 3. Results

### 3.1. Determination of optimal fermentation conditions for Eucommia leaf sweet rice wine

**Figure 1:**
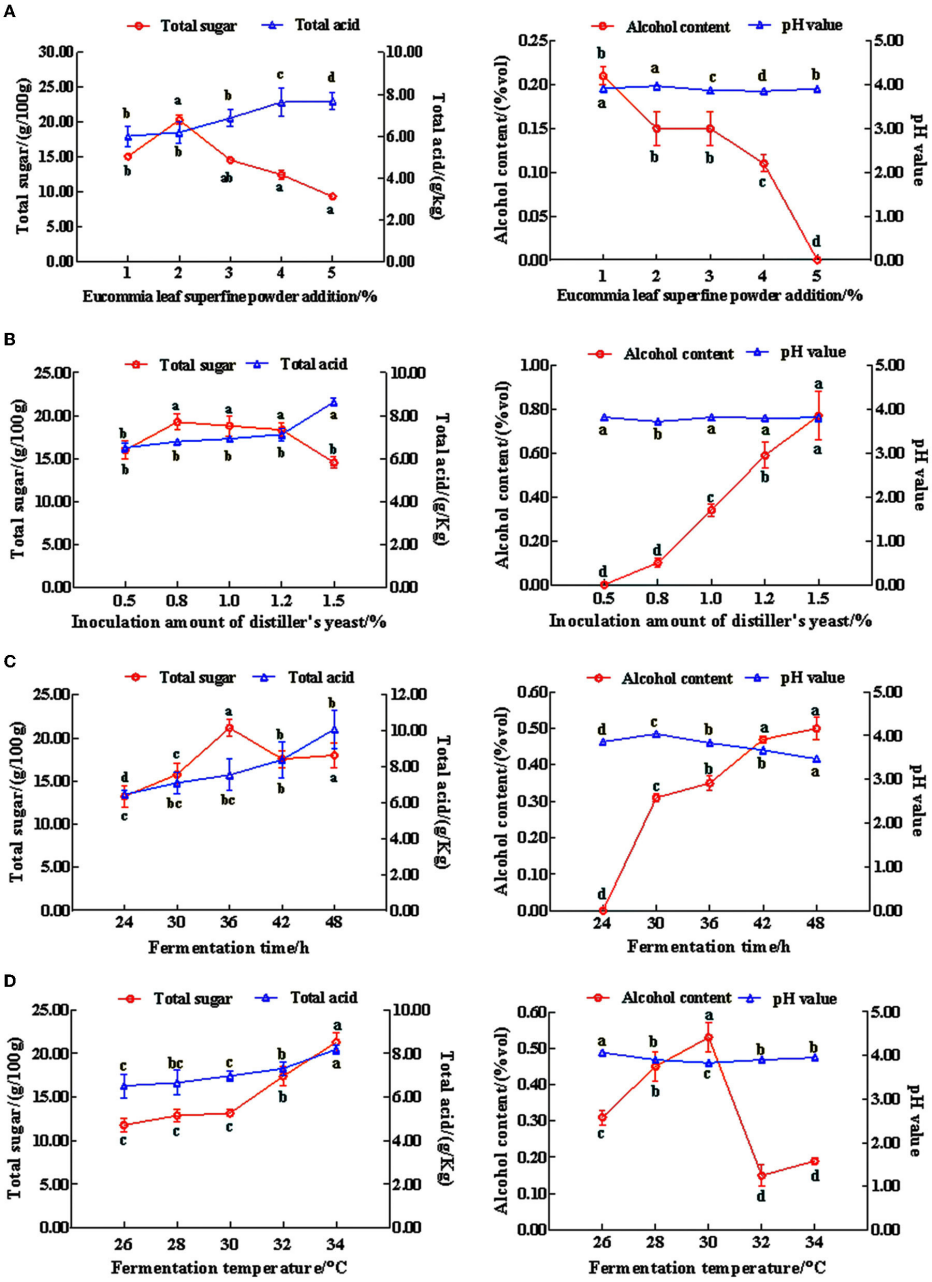
The effects on the total sugar and total acid, alcohol content, and pH value of mixed sweet rice wine. **(A)** Amount of Eucommia leaf superfine powder; **(B)** inoculation amount of distiller's yeast; **(C)** fermentation time; **(D)** fermentation temperature (*n* = 3). Different lowercase letters indicate significant differences (*p* < 0.05).

According to the results of the single-factor (Supplementary Tables 1–4) and orthogonal experiments (Supplementary Table 5), our verification results suggested that the sensory score of the optimal fermentation conditions from the orthogonal experiment was higher than that in the single-factor experiment (Supplementary Table 6). Therefore, the optimal fermentation conditions of Eucommia leaf sweet rice wine were obtained using orthogonal experiments, where the fermentation temperature was 32°C, the fermentation time was 36 h, the amount of koji added was 1% (g), and the amount of *Eucommia ulmoides* leaf superfine powder added was 2% (g). Using this formulation, we were able to create a sweet and mellow taste in Eucommia leaf sweet rice wine with a harmonious flavor that combines the taste of the wine with the taste of the *Eucommia ulmoides* leaves. The wine had a clear, transparent, and lustrous appearance with a light green color. It had a uniform texture and a good taste, with moderate sweetness and sourness. Moreover, the ratio of total sugar to total acid was appropriate (Figure 1). Subsequent experiments were carried out on the Eucommia leaf sweet rice wine using the same process.

In addition, the total number of colonies in Eucommia leaf sweet rice wine was 89 CFU/g, and the numbers of *Escherichia coli* and *Staphylococcus aureus* were both below the detection limit of 10 CFU/g; *Salmonella* and aflatoxin B1 were not detected, indicating that the hygiene indicators of Eucommia leaf sweet rice wine all met the national safety standards for fermented sweet rice wine (Supplementary Table 7).

### 3.2. Metabolomics analysis

**Figure 2:**
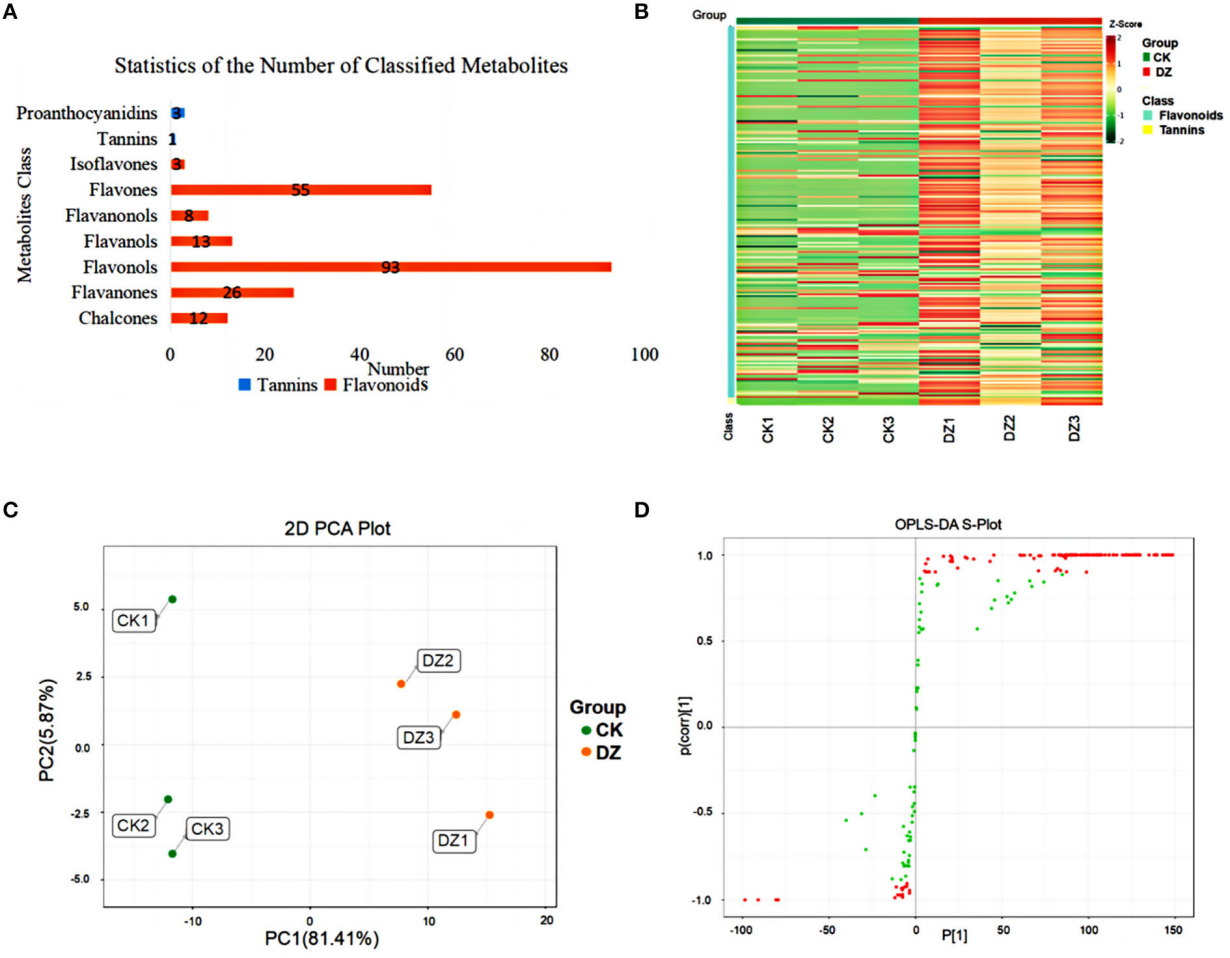
Metabolites analysis of the traditional sweet rice wine (CK) and new product (DZ). **(A)** Statistics of the number of classified metabolites; **(B)** overall sample content map, red represents high content, and green represents low content; **(C)** PCA of metabolites, and **(D)** S-plot generated from the OPLS-DA modle for CK vs. DZ. Green and red spots indicate all VMs involved in the model, and red spots suggest the most significant changes and higher contributions to the classification pattern.

An analysis of the total ion chromatogram (TIC) of different quality control (QC) samples using mass spectrometry revealed that the ion peaks of various substances in the repeated samples overlapped well, indicating that the sample signal was stable (Supplementary Figure 1). A total of 214 metabolites, mainly flavonoids and tannins, were detected in this experiment (Figure 2A).

From the cluster heatmap analysis of the two groups of samples (Figure 2B), it can be seen that the two groups of samples were significantly different, and the components of the parallel samples in the two groups were close, which proved the reliability of the samples. PCA was used to represent the differences in metabolites between traditional sweet rice wine (CK) and Eucommia leaf sweet rice wine (DZ). The six samples from the two types were classified into two different regions of the PCA plots (Figure 2C), indicating that the flavonoid metabolites of the two different types of samples were significantly different. In addition, the orthogonal partial least squares discrimination analysis (OPLS-DA) model of the two groups of samples was verified using 200 permutations. The results demonstrated that the OPLS-DA model distinguished CK and DZ to the greatest extent (R^2^X = 0.84, R^2^Y = 1, Q^2^ = 0.994), and the Q^2^ value was greater than 0.9, illustrating that the model was stable and accurate. Furthermore, the S-plot of the OPLS-DA model revealed the differences in metabolites between samples (Figure 2D).

**Figure 3:**
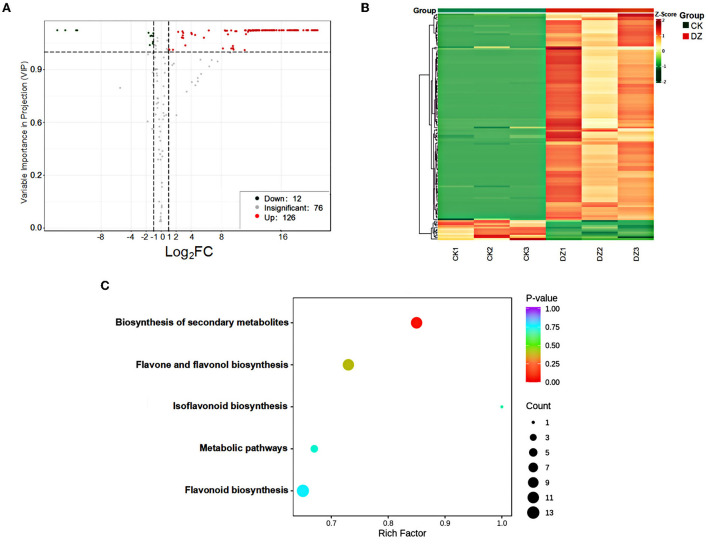
Metabolic analysis of the traditional sweet rice wine (CK) and new product (DZ). **(A)** Volcano plot; red represents upregulated metabolites, green represents downregulated metabolites, and gray represents metabolites detected but not significantly different; **(B)** heatmap and cluster analysis of different sweet rice wine samples at the metabolome level. Green indicates low abundance, and red indicates high abundance; **(C)** KEGG analysis of the differential metabolites in the sweet rice wine samples. The x-axis represents the richness factor. The color and size of the dots represents *p*-value and the amount of enriched differential metabolites, respectively.

The variable importance in projection (VIP) value was obtained from the OPLS-DA model. Based on the criteria of VIP ≥ 1 and fold change ≥ 2 or fold change ≤ 0.5, 138 differential metabolites were identified. Moreover, 126 and 12 metabolites were upregulated and downregulated in the DZ, respectively (Figure 3A). These metabolites were classified into two broad categories: flavonoids and tannins, which specifically included 68 flavonols, 25 flavonoids, 16 flavanones, 10 flavonols, 7 chalcones, 6 flavanonols, 3 proanthocyanidins, 2 isoflavones, and 1 tannin. A heatmap was used to visualize the content changes of different metabolites in the DZ (Figure 3B). The red box indicates that the metabolite content was higher than the average level in the sample, while the green box indicates that the metabolite level was lower. From the figure, it can be clearly seen that the content of flavonoid metabolites in the DZ was significantly higher than that of CK.

KEGG was used to perform pathway enrichment analysis on the metabolic pathways involved in the differential metabolites, and it was found that 40 differential metabolites could be annotated to the existing pathways in the database, and they were mainly enriched in five pathways: biosynthesis of secondary metabolites, biosynthesis of flavonoids and flavonols, biosynthesis of isoflavones, biosynthesis of flavonoids, and metabolic pathways, especially the biosynthesis of secondary metabolites (Figure 3C). A total of 11 differential metabolites were annotated to this pathway, namely, phloridzin, naringenin, kaempferol-3-O-glucoside, kaempferol-3-O-rutinoside, quercetin-3-O-glucose glycosides, rutin, catechin, gallocatechin, epigallocatechin, epicatechin, and phloretin, among which the content of quercetin-3-O-glucoside ranked second in the content of upregulated substances. The top ten metabolites with increased content were mainly quercetin compounds, in addition to hesperetin and kaempferol compounds, all of which have antibacterial and antioxidant biological properties. Therefore, it is speculated that the accumulation of quercetin, hesperetin, and kaempferol compounds is the reason for the stronger antioxidant activity of the new product in this experiment.

### 3.3. Analysis of main nutrients and functional components in the new product

**Figure 4:**
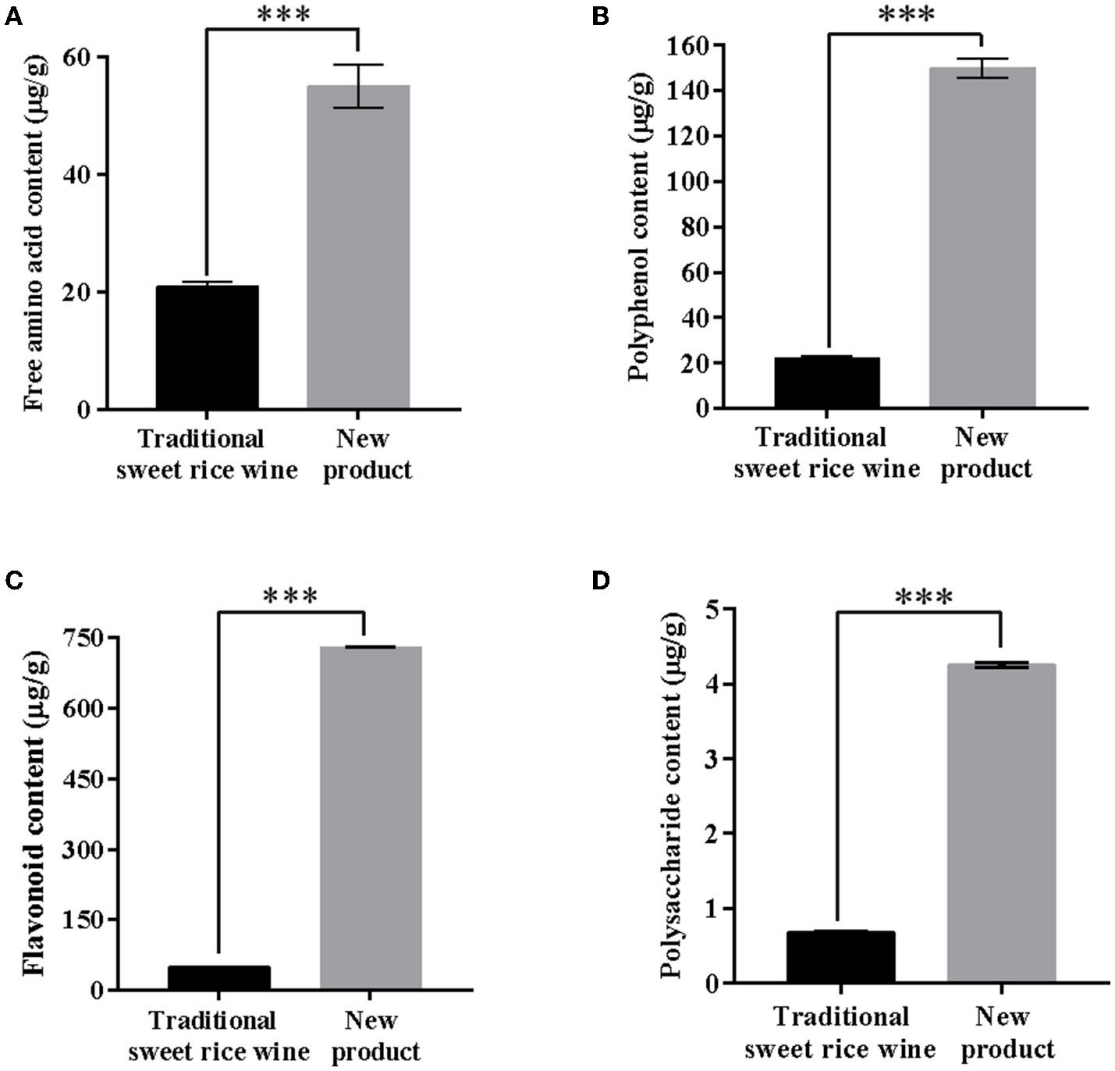
Analysis of the content of main nutrients in the new product and traditional sweet rice wine. **(A)** Free amino acid content; **(B)** polyphenol content; **(C)** flavonoid content; **(D)** polysaccharide content (*n* = 3). ***p < 0.001.

Free amino acid, polyphenol, flavonoid, and polysaccharide contents were determined. The contents were 21.0 ± 0.71 μg/g and 55.0 ± 0.37 μg/g, 22 ± 0.32 μg/g and 150 ± 0.43 μg/g, 49.6 ± 0.30 μg/g and 729 ± 0.11 μg/g, and 0.68 ± 0.02 μg/g and 4.25 ± 0.03 μg/g in the traditional sweet rice wine and new product, respectively (Figure 4). The contents of free amino acids, polyphenols, flavonoids, and polysaccharides in the new product were 2.6 times, 6.8 times, 14.7 times, and 6.3 times higher than those of traditional sweet rice wine, respectively. Indeed, it can be clearly seen that the content of flavonoids in the new product and the traditional sweet rice wine had the most significant difference (*p* < 0.001), and the content of flavonoids in the new product was much higher than that of the traditional sweet rice wine, which was consistent with the results of the metabolic analysis and indicated that the new product had higher nutritional value.

**Figure 5:**
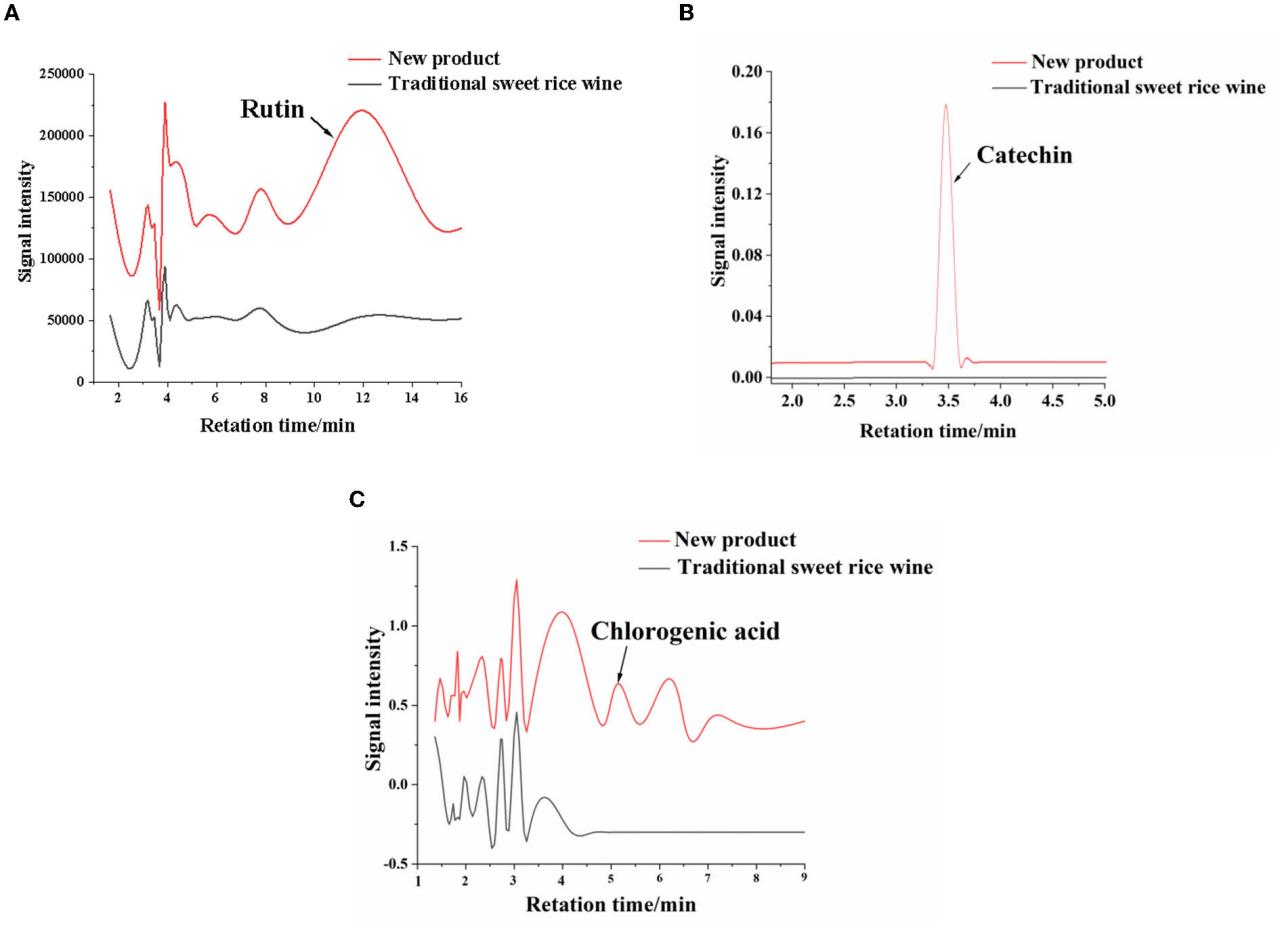
Chromatograms of functional ingredients in the new product and traditonal sweet rice wine. **(A)** Rutin; **(B)** catechin; **(C)** chlorogenic acid.

Chlorogenic acid, rutin, and catechin are important functional components in *Eucommia ulmoides* leaves, with clear antioxidant effects. The three components were determined using high-performance liquid chromatography, and the chromatograms of the standards were obtained (Supplementary Figure 2). As shown in Figure 5, the chromatogram revealed that chlorogenic acid and catechin were not detected in the traditional sweet rice wine. For catechins, people pay the most attention to tea leaves. However, catechins were detected in the new product, and the content was 1.99 ± 0.03 μg/g. The content of rutin was 4.77 ± 0.08 μg/g in the new product, while it was only 0.04 ± 0.01 μg/g in the traditional sweet rice wine. The content of chlorogenic acid was 0.90 ± 0.01 μg/g. Therefore, the contents of chlorogenic acid, rutin, and catechin in the new product were increased by adding *Eucommia ulmoides* leaf superfine powder, and the nutritional value of the new product was thus improved.

### 3.4. Antioxidant capacity of the new product *in vitro*

**Figure 6:**
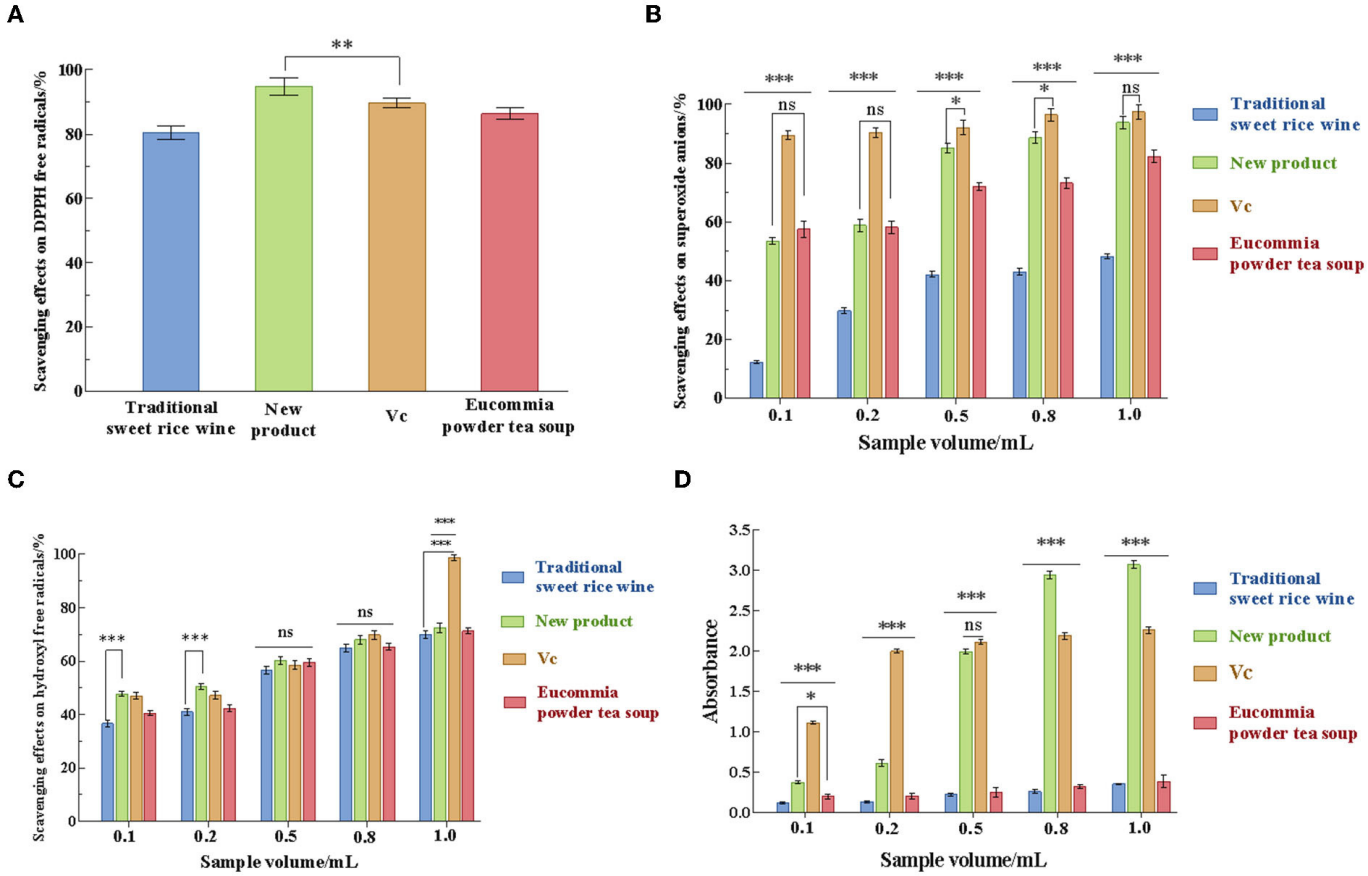
Analysis of the antioxidant activity of traditional sweet rice wine, new product, Vc, and *Eucommia ulmoides* powder tea soup. **(A)** Scavenging effects on DPPH free radicals; **(B)** scavenging effects on superoxide anions; **(C)** scavenging effects on hydroxyl free radicals; and **(D)** reduction effects. (*n* = 3). *p < 0.05; **p < 0.01; ***p < 0.001.

The *in vitro* antioxidant properties of the new product were evaluated from four aspects: DPPH free radical, superoxide anion (O2-·), hydroxyl radical (·OH) scavenging rate, and iron-reducing ability. As shown in Figure 6, Eucommia powder tea soup, the new product, traditional sweet rice wine, and ascorbic acid (Vc) all exhibited scavenging ability for those free radicals. It can be seen from the figure that, with the increase in the sample amount, the scavenging effects of Eucommia leaf sweet rice wine on DPPH, superoxide anion, and hydroxyl radicals increased significantly, showing a certain dose-effect relationship. Among the four samples, the new product had the highest DPPH free radical scavenging ability, which was 94.80 ± 0.79% (Figure 6A). When the sample amount of the new product was in the range of 0.1 mL to 0.5 mL, the scavenging effect of O2-·increased significantly with increasing sample amount (*p* < 0.001). When the sample amount was 1 mL, the scavenging rates of O2-· and ·OH reached 93.86 ± 1.14% and 72.45 ± 1.22%, respectively (Figures 6B, C). Moreover, when the amount of sample added was >0.5 mL, the reducing power of the new product was >2 mg/mL Vc. When the sample volume was 1.0 mL, the reducing power reached 1.36 times that of Vc (*p* < 0.001), indicating that the new product had effective reducing power (Figure 6D). The above results demonstrated that the new product had strong antioxidant activity.

### 3.5. α-glucosidase and α-amylase inhibition capacity *in vitro*

**Figure 7:**
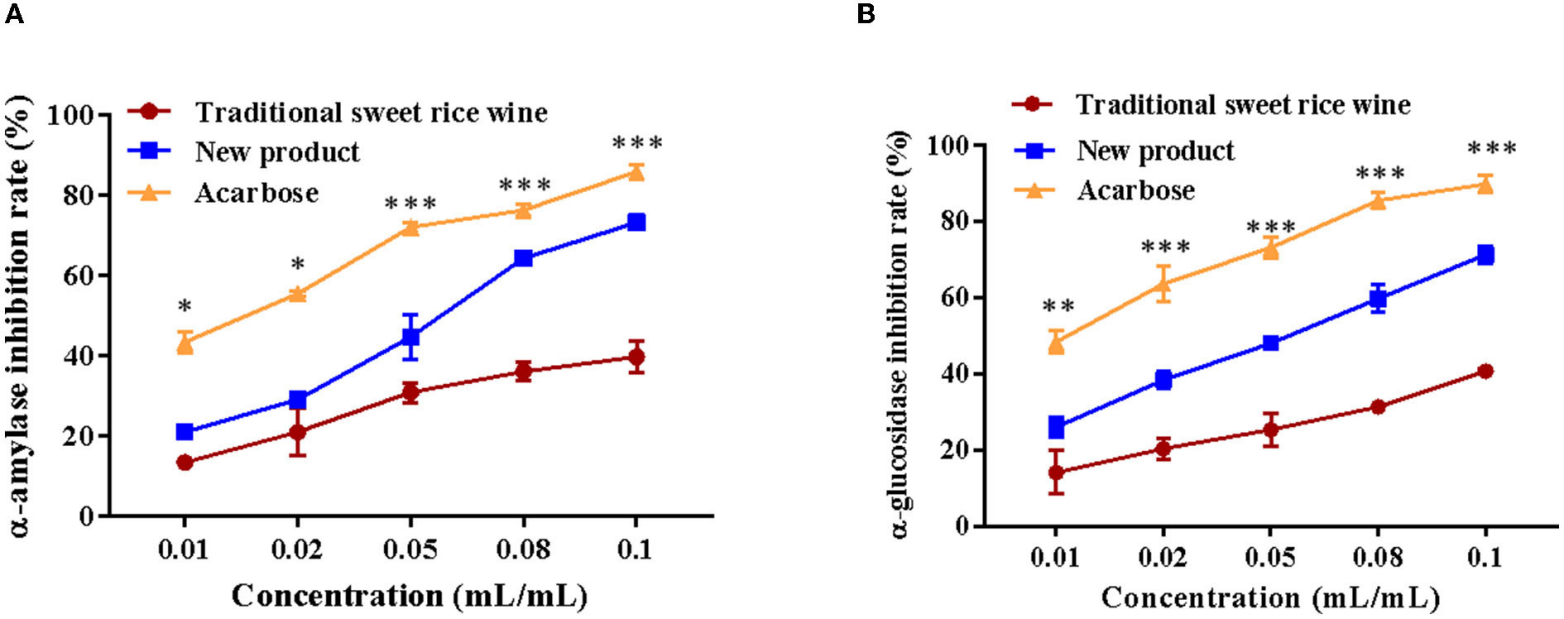
Inhibitory effects on α-amylase **(A)** and α-glucosidase **(B)** of traditional sweet rice wine, new product and acarbose (*n* = 3). **p < 0.01; ***p < 0.001.

The inhibitory activities of α-glucosidase and α-amylase *in vitro* of traditional sweet rice wine and Eucommia leaf sweet rice wine were determined. Acarbose was used as a positive control. As shown in Figure 7, both traditional sweet rice wine and Eucommia leaf sweet rice wine suggested inhibitory effects on the two enzymes, but the inhibition of Eucommia leaf sweet rice wine was significantly higher than that exhibited by traditional sweet rice wine (*p* < 0.001). With increasing sample concentrations, the inhibitory rate continued to increase, showing a dose–effect relationship. When the sample volume concentration reached 0.1 mL/mL, the inhibition rates of α-glucosidase in traditional sweet rice wine and Eucommia leaf sweet rice wine were 40.66 ± 0.51% and 71.24 ± 2.20%, respectively, which were 45.34 and 79.8% of acarbose (89.67 ± 6.18%), and the inhibition rates of α-amylase were 39.68 ± 3.82% and 73.23 ± 1.89%, respectively, which were 46.23 and 85.3% of acarbose (85.84 ± 1.80%). The results demonstrated that Eucommia leaf sweet rice wine could effectively inhibit α-glucosidase and α-amylase *in vitro*.

### 3.6. Cholate binding ability *in vitro*

**Figure 8:**
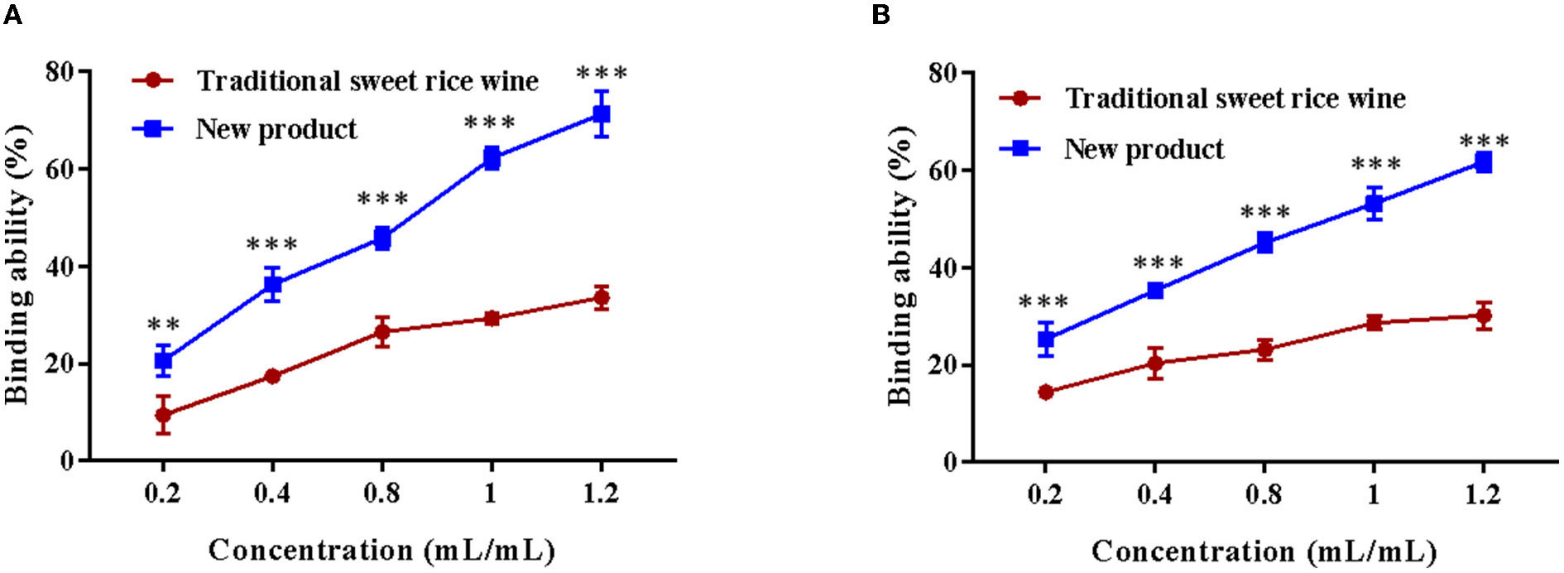
Cholate binding ability of traditional sweet rice wine and new product. **(A)** Sodium glycocholate binding capacity, **(B)** sodium taurocholate binding capacity (*n* = 3). **p < 0.01; ***p < 0.001.

Sodium glycocholate and sodium taurocholate were selected as the binding objects to analyze the binding ability of Eucommia leaf sweet rice wine and evaluate the hypolipidemic effect *in vitro*. As shown in Figure 8, the sample volume concentration was positively correlated with the binding capacity of cholate. With an increase in the concentration of the new product, the binding rate of sodium glycocholate and sodium taurocholate increased significantly (*p* < 0.001), and the binding capacity of sodium glycocholate was relatively high, while with the increase in the concentration of the traditional sweet rice wine, the binding rate of sodium glycocholate gradually became saturated. When the sample concentration was 1.2 mL/mL, the binding ability of Eucommia leaf sweet rice wine to sodium glycocholate (71.25 ± 4.56%) and sodium taurocholate (61.67 ± 1.92%) was 2.12 and 2.05 times that of traditional sweet rice wine, respectively.

### 3.7. ACE inhibition ability *in vitro*

**Figure 9:**
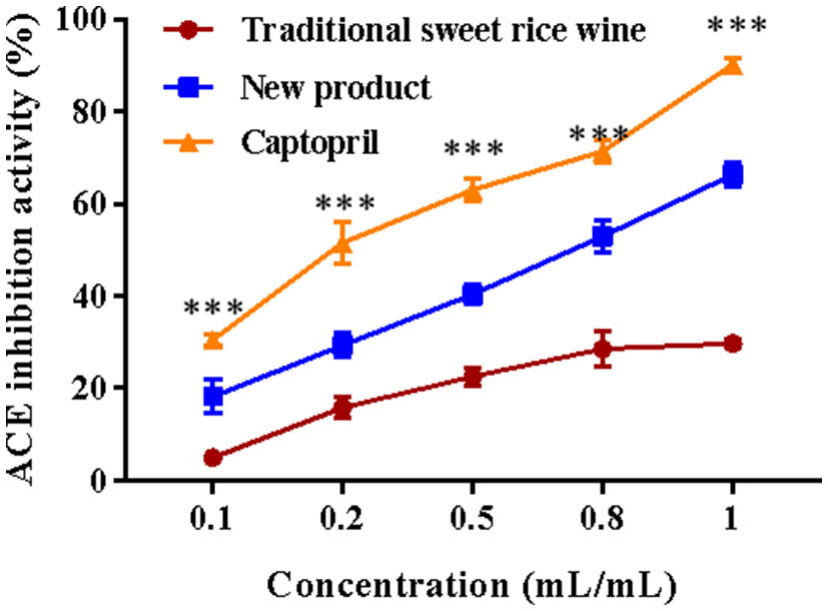
Angiotensin-converting enzyme (ACE) inhibitory activity of traditional sweet rice wine, new product, and captopril (*n* = 3). ***p < 0.001.

The ACE inhibitory activity of traditional sweet rice wine, Eucommia leaf sweet rice wine, and the drug control captopril were evaluated *in vitro*. As shown in Figure 9, the ACE inhibition rate increased with sample and drug concentrations. For traditional sweet rice wine, the increasing trend of ACE inhibition activity was not clear when the concentration ranged from 0.5 to 1 mL/mL. With increasing concentration, the inhibitory activity of Eucommia leaf sweet rice wine on ACE increased significantly (*p* < 0.001), which exhibited a strong inhibition rate, although it was lower than that of captopril. When the concentration was 1 mL/mL, the inhibitory activity of Eucommia leaf sweet rice wine on ACE reached 66.32 ± 6.71%, which was 73.58% of the inhibition rate of captopril (90.13 ± 1.49%), indicating that Eucommia leaf sweet rice wine had a certain role in hypotension.

## 4. Discussion

In recent years, a large number of articles have reported the chemical composition, functional substances, and pharmacological effects of *Eucommia ulmoides* Oliv. (30). The bark of *Eucommia ulmoides* Oliv. is used rather than the leaves. However, the primary components and pharmacological effects of *Eucommia ulmoides* leaves are comparable to those of *Eucommia ulmoides* bark, and the antioxidant capacity of *Eucommia ulmoides* leaves is even higher than that of *Eucommia ulmoides* bark (31, 32). Therefore, the product development of *Eucommia ulmoides* leaves has good prospects. In this study, a new healthcare product, Eucommia leaf sweet rice wine, was developed. The Eucommia leaf sweet rice wine fermentation procedure was optimized to maximize the retention of the wine flavor, fragrance, and antioxidant components of *Eucommia ulmoides* leaves. Ultimately, a fermentation temperature of 32°C, a fermentation time of 36 h, an additional amount of koji of 1% (g), and Eucommia superfine leaf powder of 2% (g) were determined to be the best conditions for the fermentation process. This process yields sweet rice wine with a special flavor. The total number of colonies of *Escherichia coli, Staphylococcus aureus*, and other health indicators in the new product all reached the national standards for fermented wine.

Flavonoids are a large class of polyphenol oxides and a class of structurally diverse secondary metabolites (33, 34). Numerous pharmacological studies demonstrated the antioxidant, anticancer, and anti-inflammatory functions of flavonoids (35). Plant metabolomic analysis allows us to study the relationship between metabolites produced by biological processes and plant properties (36). Li et al. used UPLC-MS to analyze the metabolites in the different parts of *Eucommia ulmoides* Oliv. and found a large number of flavonoids in the *Eucommia ulmoides* leaves (37). The findings of this study were consistent with this conclusion; 126 flavonoids with upregulated contents were determined in the metabolites of the new product, among which quercetins had the highest content, such as isoquercitrin, spiral side, hyperin, quercetin-7-O-glucoside, quercetin-7-O-rutinoside, and quercetin-3-O-sambubioside. Quercetin compounds are considered natural for cancer treatment (38, 39).

Additionally, the flavonoids with relatively different contents in the new product are also kaempferol, including nicotiflorin and astragalin. Studies found that nicotiflorin has protective effects against memory dysfunction, energy metabolism failure, and oxidative stress in patients with multiple cerebral infarctions (40). As a bioactive phytochemical with potential therapeutic activity, astragalin has anti-inflammatory, antioxidant, neuroprotective, cardio-protective, anticancer, antiulcer, and antidiabetic properties, and its mechanism of action as a candidate drug has been studied by an increasing number of people in the past 2 years (41). These results indicated that, after the optimal fermentation process, the Eucommia leaf sweet rice wine retained a large number of active substances with antioxidant properties.

In recent years, numerous studies showed that the enhancement of free radical activity causes many clinical diseases and that the decrease in the body's antioxidant capacity is mostly due to the increase in the number of free radicals in the body. *Eucommia ulmoides* leaves contain a variety of antioxidant components, which can be developed as natural plant antioxidants and are also a hot topic in food and medicine research at present (42). In this study, DPPH, O2-, ·OH, and FRAP were used to determine the free radical scavenging capacity of the new product. The new product showed high antioxidant activity, and the DPPH free radical scavenging capacity and iron-reducing capacity were higher than those of the antioxidant Vc. The scavenging rates of O2-· and ·OH were lower than that of Vc; however, the new product also presented strong scavenging rates as a kind of food. Phenolic compounds, especially flavonoids, are compounds associated with the antioxidant capacity of plant extracts. Studies showed that the total phenol content in *Eucommia ulmoides* leaves correlates well with its antioxidant activity (43). Liu et al. extracted polysaccharides from *Eucommia ulmoides* leaves and found that they had significant scavenging effects on DPPH free radicals, OH free radicals, and ABTS free radicals (44). The results of this study were consistent with the above conclusions. The new product contains a high concentration of flavonoids, and its strong antioxidant activity is mainly attributed to the presence of flavonoids, polysaccharides, polyphenols, rutin, and other compounds.

Currently, triple H (hypertension, hyperglycemia, and hyperlipidemia) has become a major health management problem throughout society. The inhibition of digestive enzyme (α-amylase and α-glucosidase) activity is an important way to control hyperglycemia. As a large category of nonessential dietary ingredients, phenolic substances have many pharmacological effects, such as antioxidant, antimutagenic, anti-inflammatory, and anticancer properties, as well as chelating digestive enzymes (45–47). In addition, studies found that quercetin and rutin can reduce the risk of diabetes (48–50). In this study, the contents of polyphenol and rutin in the new product were 150 ± 0.43 μg/g and 4.77 ± 0.08 μg/g, respectively, and many upregulated quercetin compounds are also shown in the new product. An inhibition experiment with digestive enzymes suggested that Eucommia leaf sweet rice wine had effective inhibitory activities on α-glucosidase and α-amylase. At a concentration of 0.1 mL/mL, the inhibitory rates were 71.24 ± 2.20% and 73.23 ± 1.89%, respectively, which reached 79.8% and 85.3% of acarbose, indicating that it had high hypoglycemic activity. This conclusion confirms that the high content of polyphenols, quercetin, and rutin in the Eucommia leaf sweet rice wine may be the reason for its strong antihyperglycemic effect.

Moreover, polyphenols can reduce hyperlipidemia by binding with bile acids, and flavonoids could decrease cholesterol, free fatty acids, and low-density lipoprotein, indicating that the latter also greatly modulates the effect on hyperlipidemia (51–53). The results of this study demonstrated that the flavonoid content in the new product was as high as 729 ± 0.11 μg/g, which was 14.7 times that of traditional sweet rice wine. In the antihyperlipidemic experiment, Eucommia leaf sweet rice wine has shown a positive binding capacity for both sodium glycocholate and sodium taurocholate, with a higher binding capacity for sodium glycocholate. At a concentration of 1.2 mL/mL, the binding capacity reached 71.25 ± 4.56%, which was much higher than that of traditional sweet rice wine (33.59 ± 2.31%). This phenomenon may be related to the high content of polyphenols and flavonoids in *Eucommia ulmoides* leaves. ACE is a key enzyme that can cause hypertension symptoms and lead to various diseases (54). Inhibition of ACE can effectively reduce hypertension. Flavonoids and chlorogenic acid in *Eucommia ulmoides* leaves can effectively reduce hypertension (55). The chlorogenic acid content in the new product was 0.90 ± 0.01 μg/g, while it was not detected in the traditional sweet rice wine. This study suggested that Eucommia leaf sweet rice wine had a certain inhibitory effect on the ACE enzyme, which was lower than that of the drug control captopril but certainly higher than that of the traditional sweet rice wine.

In conclusion, our results proved that flavonoids and polyphenols are abundant components in Eucommia leaf sweet rice wine. The existence of these components provides Eucommia leaf sweet rice wine with strong antioxidant activity and positive hypoglycemic, hypolipidemic, and hypotensive effects.

## 5. Patent

*Eucommia ulmoides* leaf glutinous rice sweet wine and preparation method.: YZ, WG, YX, and D-gZ (Application No.: CN202110645889.9).

## Data availability statement

The original contributions presented in the study are included in the article/Supplementary material, further inquiries can be directed to the corresponding authors.

## Author contributions

Conceptualization and methodology: YZ, WG, and NR. Investigation: NR, WG, and YX. Writing—original draft: NR and YZ. Project administration and writing—review and editing: YZ and D-gZ. All authors contributed to the manuscript and approved the submitted version.
