# Supplementary material for: Innovation in sweet rice wine with high antioxidant activity: Eucommia ulmoides leaf sweet rice wine
Source: Front Nutr. 2023 Jan 10;9:1108843. doi: 10.3389/fnut.2022.1108843 (PMC9871602; doi:10.3389/fnut.2022.1108843)
Supplement: Supplementary file 1 [file Data_Sheet_1.docx]

Supplementary Material

# Method

- 1. Single-Factor Experiment and Orthogonal Experiment on Fermentation Conditions

1. Amount of *Eucommia ulmoides* leaf superfine powder added: The amount of koji added was 1 %, and the product was fermented at 30 ℃ for 36 h. The effect of the *Eucommia ulmoides* leaf superfine powder addition (1 %, 2 %, 3 %, 4 %, 5 %) on the fermentation of the new product was investigated. The total sugar, total acid, alcohol content, and pH value were determined (the same below).
2. Koji inoculation amount: *Eucommia ulmoides* leaf superfine powder was added at a level of 2 %, and the product was fermented at 30 °C for 36 h. The influence of the addition of koji (0.5 %, 0.8 %, 1.0 %, 1.2 %, 1.5 %) on the fermentation of the mixed sweet wine was investigated.
3. Fermentation time: The amount of *Eucommia ulmoides* leaf superfine powder added was 2 %, the added amount of koji was 0.8 %, and the fermentation temperature was 30 °C. The effect of the fermentation time (24 h, 30 h, 36 h, 42 h, 48 h) on the fermentation of the mixed sweet wine was investigated.
4. Fermentation temperature: The amount of *Eucommia ulmoides* leaf superfine powder added was 2 %, the amount of koji added was 0.8 %, and the fermentation time was 36 h. The influence of the fermentation temperature (26 °C, 28 °C, 30 °C, 32 °C, 34 °C) on the fermentation of the mixed sweet wine was investigated.

On the basis of a single-factor experiment, the four factors of fermentation temperature (A), fermentation time (B), koji inoculation amount (C), and *Eucommia ulmoides* leaf superfine powder amount (D) were selected, and the L_9_ (4^3^) orthogonal experiment was designed.

- 1. Determination of Total Soluble Sugars (Anthrone Sulfate Colorimetric Method)

Anhydrous glucose was used to prepare 200, 150, 100, 50, and 25 µg/mL standard glucose aqueous solutions. We drew 1.0 mL volumes of different concentrations of standard glucose droplets into the volumetric flasks that were pre-filled with 8.0 mL of anthrone reagent and shook them while dripping. Water was used as a blank control. It was heated accurately in a boiling water bath for 7 min, after which we immediately took it out and placed it in an ice bath to cool it to room temperature, transferred it into a 10 mm cuvette, and then measured the absorbance at a wavelength of 620 nm. Drawing the standard curve with the concentration as the abscissa and the absorbance as the ordinate, the regression equation was obtained. Then, we weighed 1 g of both the new product and the traditional sweet wine (accurate to 0.0002 g), added 80 mL of boiling water, extracted the mixture in a boiling water bath for 30 min, then filtered it immediately. We washed the residue with boiling water several times, combined the filtrates, added water to bring the volume up to 500 mL, and shook well for later use. Then, we took 3 dried volumetric flasks and accurately transferred 8.0 mL of anthrone reagent into each. At this point, 1.0 mL of the prepared sample solution dropwise was added dropwise to volumetric flasks No. 1-2 and 1.0 mL of distilled water was added dropwise to volumetric flask No. 3. The flasks were shaken well and we placed them in a boiling water bath for accurate heating for 7 min. Then, we immediately took them out and placed them in an ice bath to cool down to room temperature, transferred them to a 10 mm cuvette, and measured their absorbance at a 620 nm wavelength.

- 1. Metabolites Analysis

UPLC Conditions

Agilent SB-C18 (1.8 µm, 2.1 mm * 100 mm) was selected as the chromatographic column. The mobile phase was consisted of solvent A, pure water with 0.1 % formic acid, and solvent B, acetonitrile with 0.1 % formic acid. Sample measurements were performed with a gradient program that employed the starting conditions of 95 % A, 5 % B. Within 9 min, a linear gradient to 5 % A, 95 % B was programmed, and a composition of 5 % A, 95 % B was kept for 1 min. Subsequently, a composition of 95 % A, 5.0 % B was adjusted within 1.1 min and kept for 2.9 min. The flow velocity was set as 0.35 mL per minute; The column oven was set to 40 ℃; The injection volume was 4 μL. The effluent was alternatively connected to an ESI-triple quadrupole-linear ion trap (QTRAP)-MS.

ESI-Q TRAP-MS/MS

The ESI source operation parameters were as follows: source temperature 550 ℃; ion spray voltage (IS) 5500 V (positive ion mode)/-4500 V (negative ion mode); ion source gas I (GSI), gas II (GSII), curtain gas (CUR) were set at 50, 60, and 25 psi, respectively; the collision-activated dissociation (CAD) was high. Instrument tuning and masscalibration were performed with 10 and 100 μmol/L polypropylene glycol solutions in QQQ and LIT modes, respectively. QQQ scans were acquired as MRM experiments with collision gas (nitrogen) set to medium. DP (declustering potential) and CE (collision energy) for individual MRM transitions was done with further DP and CE optimization. A specific set of MRM transitions were monitored for each period according to the metabolites eluted within this period.

- 1. Detection of Flavonoids Content

1. Preparation of the standard substance (reference substance) solution: A total of 10 mg of rutin reference substance was weighed precisely in a 50 mL volumetric flask, 70 % ethanol was added, and the mixture was extracted and dissolved by ultrasonic waves. After this, we let the mixture cool, added 70 % ethanol up to the mark, and shook it evenly to obtain 0.2 mg/mL rutin standard.

b. Preparation of standard curve: Amounts of 0, 0.125, 0.625, 1.25, 3.125, and 6.25 mL of the reference solution were drawn into 25 mL volumetric flasks. Then, we added 6 mL of water, added 1 mL of 5 % sodium nitrite, shook the mixture evenly, and let it stand at room temperature. After 6 min, we added 1 mL of 10 % aluminum nitrate solution, shook the mixture evenly, let it stand at room temperature for 6 min, added 10 mL of 4 % sodium hydroxide solution, added water up to the mark, let it stand for 15 min. The corresponding solvent was used as a blank, and we measured the absorbance at a wavelength of 510 nm. The abscissa represented the concentration, the ordinate represented the absorbance, and a standard curve was drawn.

c. Sample preparation: A total of 4.0000 g of *Eucommia ulmoides* leaf superfine powder, traditional sweet rice wine, and new products were weighed and put into a conical flask with a stopper. Then, we added 30 mL of 70 % ethanol precisely, extracted the mixture using ultrasonic waves for 20 min, let it cool to room temperature, and weighed it again. The lost mass was supplemented with 70 % ethanol, then the mixture was shaken and concentrated to 1 mL with a rotary evaporator. We took 1 mL of the concentrated solution and measured the absorbance according to the standard curve preparation method.

1.5 Determination of Total Polysaccharide Content

a. Preparation of standard substance (reference substance) solution: A total of 0.1 g of glucose was weighed in a 100 mL volumetric flask and we diluted it to the mark with distilled water to obtain 1 mg/mL of total glucose reference substance solution. We then precisely measured out 1 mL of 1 mg/mL glucose solution and diluted it to 10 mL to obtain 100 μg/mL of glucose solution.

b. Standard curve preparation: A pipette was used to transfer 0.01, 0.05, 0.10, 0.50, 1.00, 1.50, and 3.00 mL of 100 μg/mL glucose solution into 10 mL volumetric flasks. Then, we added 4 mL of sulfuric acid-anthrone solution to each and diluted the mixture to the mark with distilled water. We inverted and mixed the sample, brought it to room temperature, heated it in a 100 °C water bath for 10 min, then brought it back to room temperature, then measured the absorbance at 622 nm. The abscissa represented the glucose concentration, the ordinate represented the absorbance, and we drew a standard curve.

c. Sample preparation: We accurately weighed out 2.00 g of *Eucommia ulmoides* leaf superfine powder, traditional sweet glutinous rice wine, and new product. Then, 30 mL of 85 % ethanol was used as a solvent, we heated and refluxed the mixture in a Soxhlet extractor to degrease it for 2 h, filtered the mixture with suction, and washed it with 85 % ethanol 6 times. We then dried the mixture with a material–liquid ratio of 1:10, extracted it twice in a 100 °C water bath for 2 h each time, concentrated the mixture to 5 mL, and then diluted it up to the mark in a 25 mL volumetric flask as the test solution for the determination of the total polysaccharides. We accurately drew 1.0 mL of the test solution and measured the absorbance according to the standard curve preparation method.

1.6 Determination of Rutin Content

a. Standard curve preparation: A total of 2.5 mg of rutin standard was weighed out, dissolved with methanol, made up to 1 mg/mL, and stored in a 4 °C refrigerator for later use. The mixture was diluted to 100 μg/mL, 50 μg/mL, 25 μg/mL, 12.5 μg/mL, 6.25 μg/mL, and 1 μg/mL and prepared in a standard curve.

b. Sample preparation: We precisely weighed out 2 g of *Eucommia ulmoides* leaf superfine powder, traditional sweet glutinous rice wine, and the new product (accurate to 0.001 g), added anhydrous methanol solution to each sample, and ultrasonicated the mixture in an ultrasonic cleaner for 30 min. The ratio of material to liquid was 1:10. We then filtered the mixture, kept the filtrate, repeated the above steps for the residue, combined the filtrate twice, mixed it well, and used a rotary evaporator to concentrate it to 1 mL. The sample solution was centrifuged at 12,000 r/min for 20 min, and the supernatant was taken.

c. Chromatographic conditions: Mobile phase A was 0.02 % formic acid, and mobile phase B was acetonitrile. HPLC instrument gradient elution program setting conditions: 0~17 min, 20 % B, flow rate set to 1.0 mL/min, wavelength set to 360 nm, column temperature set to 25 °C, injection volume of 5 μL, and each sample injected repeatedly 3 times.

1.7 Determination of Chlorogenic Acid Content

a. Standard curve preparation: We accurately weighed 2 mg of chlorogenic acid standards, dissolved it in methanol to bring it to 1 mg/mL, and stored it in a refrigerator at 4 °C for later use. We diluted the mixture with methanol to 80 μg/mL, 40 μg/mL, 20 μg/mL, 10 μg/mL, 5 μg/mL, and 1 μg/mL, then prepared a standard curve.

b. Sample preparation: We precisely weighed out 4 g (accurate to 0.001 g) each of *Eucommia ulmoides* leaf superfine powder, traditional sweet wine, and the new product into a 10 mL volumetric flask, added 8 mL of 70 % methanol, ultrasonically extracted the mixture for 30 min, and diluted it to the mark with 70 % methanol. We mixed the solution well, concentrated it to 1 mL with a rotary evaporator, passed it through a 0.45 μm filter membrane, and used the filtrate for a liquid chromatography analysis.

c. Chromatographic conditions: Mobile phase: 0.5 % acetic acid solution: acetonitrile (V:V) = 9:1. Retention time: 10 min, with the flow rate set to 1.0 mL/min, the wavelength set to 327 nm, the column temperature set to 35 °C, the injection volume set to 10 μL, and each sample being injected 3 times. The calculation result retained three significant figures.

1.8 Determination of Catechin (EGCG) Content

a. Standard curve preparation: We accurately weighed 2 mg of EGCG standard, dissolved it in a stable solution to bring it to 1 mg/mL, and stored it in a 4 °C refrigerator for later use. We diluted the mixture with stable solution to 200 μg/mL, 100 μg/mL, 50 μg/mL, 25 μg/mL, 12.5 μg/mL, 6.25 μg/mL, and 1.0 μg/mL, and made a standard curve.

b. Sample preparation: We precisely weighed 2 g of *Eucommia ulmoides* leaf superfine powder, traditional sweet glutinous rice wine, and the new product (accurate to 0.001 g); added anhydrous methanol solution to each sample; and ultrasonicated the mixture in an ultrasonic cleaner for 30 min. The ratio of material to liquid was 1:10. We then filtered the mixture, kept the filtrate, and repeated the above steps for the residue. The ratio of material to liquid was 1:10. We filtered the solution, combined the filtrate twice, mixed it well, and used a rotary evaporator to concentrate it to 1 mL. The sample solution was centrifuged at 12,000 r/min for 20 min and the supernatant was taken. A pipette was used to transfer 2 mL of the mother solution into a 10 mL volumetric flask; then, we diluted it to the mark with a stable solution, shook it well, and passed it through a 0.45 μm membrane for measurement.

c. Chromatographic conditions: Mobile phase A: we added 90 mL of acetonitrile, 20 mL of acetic acid, and 2 mL of EDTA-2Na solution into a 1000 mL volumetric flask; diluted it to the mark with water; shook it well; and passed it through a 0.45 μm membrane. Mobile phase B: we added 800 mL of acetonitrile, 20 mL of acetic acid, and 2 mL of EDTA-2Na solution into a 1000 mL volumetric flask; diluted it to the mark with water; shook it well; and passed it through a 0.45 μm membrane for measurement. At 0~10 min: 100 % A; 10~25 min: 68 % A, 32 % B; 25~35 min: 68 % Phase A, 32 % B. The flow rate was set to 1.0 mL/min, the wavelength was set to 278 nm, the column temperature was set to 35 °C, the injection volume was 10 μL, and each sample was injected 3 times.

# Supplementary Figures and Tables

##
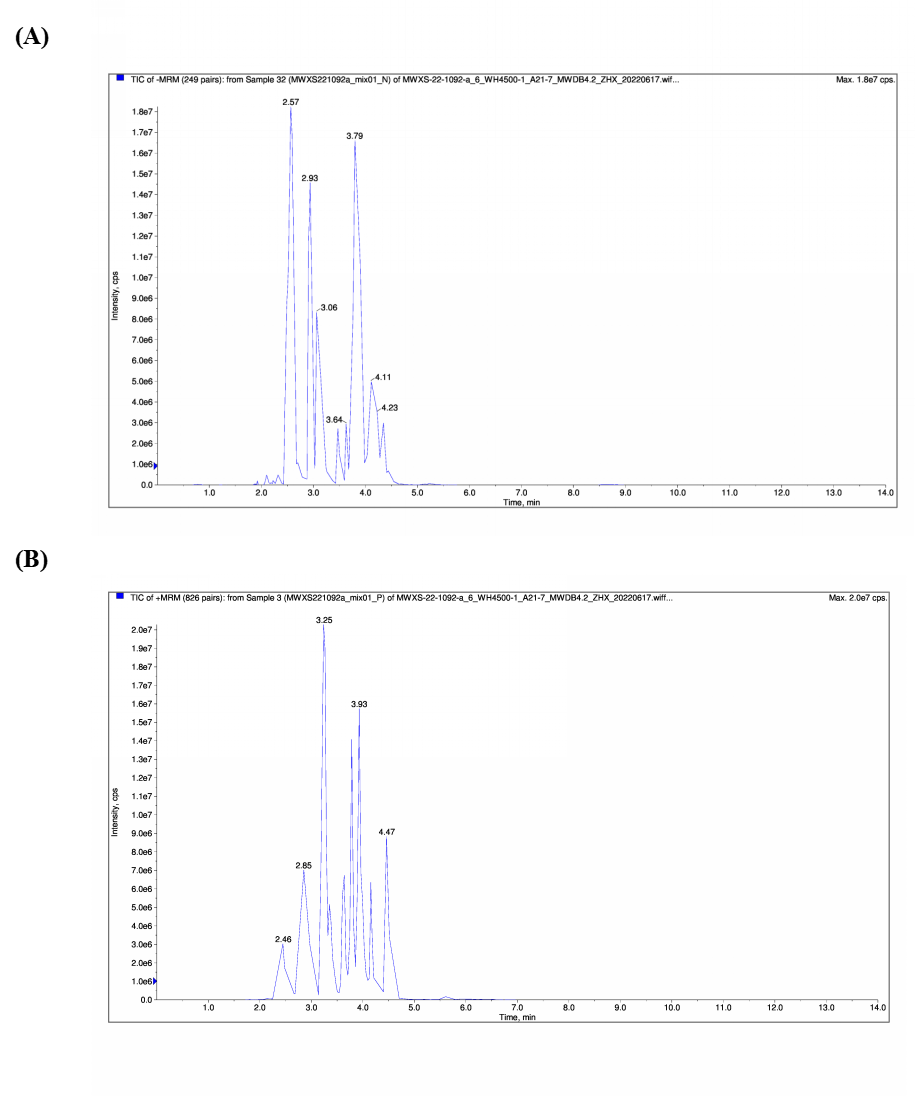
Supplementary Figures

**Figure 1.** Analysis of the total ion current (TIC) in different quality control (QC) samples. The abscissa represents the retention time (min) of metabolite detection, and the ordinate represents the intensity (cps: count per second) of the ion current.

Note: **(A)** Represents negative ion mode, **(B)** Represents positive ion mode.


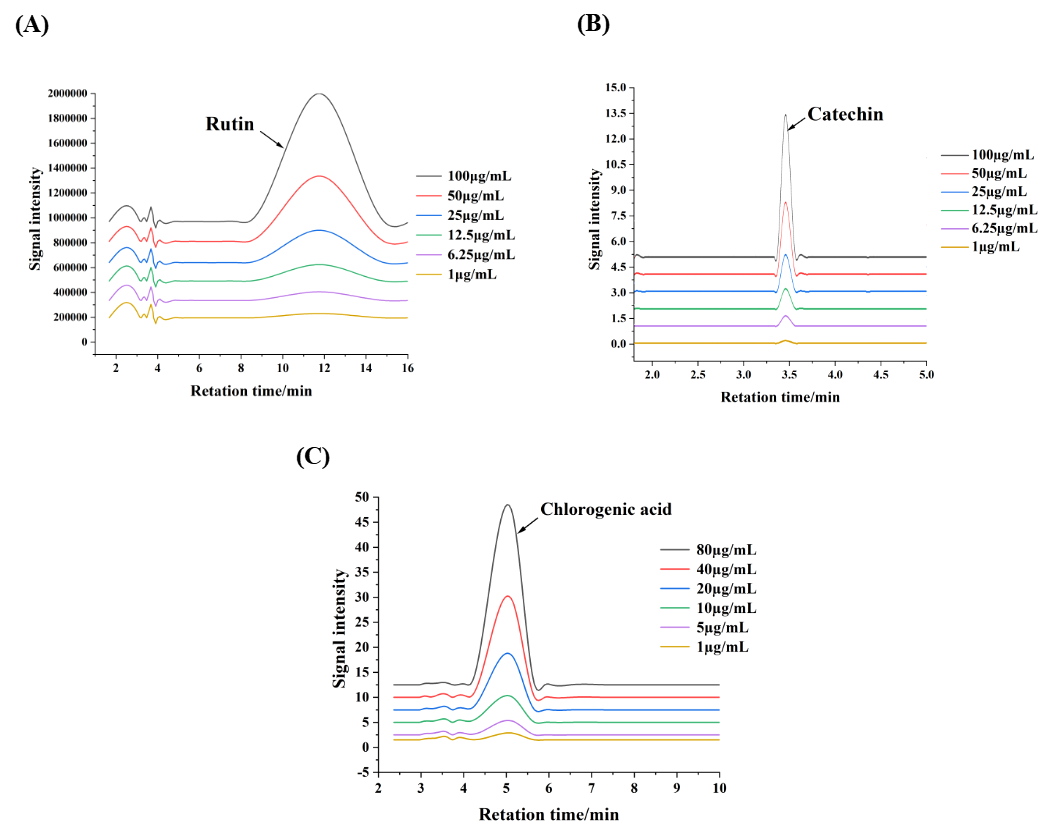


**Figure 2**. Chromatogram of chlorogenic acid (**A**), rutin (**B**), and catechin (**C**) standards.

## Supplementary Tables

**Table 1**. Sensory scores of *Eucommia ulmoides* leaves superfine powder addition.

| **Evaluation item** | **Addition amount of Eucommia leaf superfine powder/%** | | | | |
| --- | --- | --- | --- | --- | --- |
|  | **1.0** | **2.0** | **3.0** | **4.0** | **5.0** |
| Color and shape (30) | 27.71±1.2^a^ | 26.97±0.5^a^ | 25.33±0.6^b^ | 24.21±0.5^b^ | 22.70±0.3^c^ |
| Taste (40) | 36.93±0.8^a^ | 36.49±0.4^a^ | 34.14±0.4^b^ | 31.86±0.4^c^ | 30.57±0.5^c^ |
| Odor (30) | 26.14±0.8^b^ | 27.8**3**±0.5^a^ | 27.14±0.4^a^ | 25.71±0.5^c^ | 23.71±0.5^d^ |
| Total | 90.79±0.2^a^ | 91.29±0.3^a^ | 86.61±0.4^b^ | 81.79±0.6^c^ | 76.99±0.3^d^ |

**Table 2**. Sensory scores of inoculation amount of koji.

| **Evaluation item** | **Koji inoculation amount/%** | | | | |
| --- | --- | --- | --- | --- | --- |
|  | **0.5** | **0.8** | **1.0** | **1.2** | **1.5** |
| Color and shape (30) | 24.75±0.2^a^ | 24.88±0.5^a^ | 24.25±0.2^a^ | 23.00±0.3^b^ | 22.38±0.2^b^ |
| Taste (40) | 31.50±0.5^a^ | 32.75±0.4^a^ | 31.63±0.2^b^ | 31.75±0.3^b^ | 29.75±0.2^c^ |
| Odor (30) | 25.38±0.4^a^ | 25.25±0.3^a^ | 24.63±0.3^b^ | 25.38±0.4^a^ | 24.50±0.4^b^ |
| Total | 81.63±0.3^b^ | 82.88±0.3^a^ | 80.50±0.4^c^ | 80.13±0.4^c^ | 76.63±0.4^d^ |

**Table 3**. Sensory scores of fermentation time.

| **Evaluation item** | **Fermentation time/h** | | | | |
| --- | --- | --- | --- | --- | --- |
|  | **24** | **30** | **36** | **42** | **48** |
| Color and shape (30) | 23.68±0.3^b^ | 25.71±0.4^a^ | 26.14±0.2^a^ | 25.14±0.3^a^ | 25.14±0.2^a^ |
| Taste (40) | 28.14±0.3^d^ | 31.00±0.4^c^ | 33.43±0.2^a^ | 32.86±0.3^b^ | 33.14±0.3^a^ |
| Odor (30) | 24.14±0.5^b^ | 25.07±0.6^a^ | 25.71±0.6^a^ | 25.29±0.4^a^ | 25.43±0.3^a^ |
| Total | 76.14±0.3^d^ | 81.79±0.7^c^ | 85.29±0.6^a^ | 83.29±0.5^b^ | 83.71±0.5^b^ |

**Table 4.** Sensory scores of fermentation temperature.

| **Evaluation item** | **Fermentation temperature/℃** | | | | |
| --- | --- | --- | --- | --- | --- |
|  | **26** | **28** | **30** | **32** | **34** |
| Color and shape (30) | 24.80±0.5^b^ | 24.80±0.2^b^ | 25.20±0.3^b^ | 26.00±0.4^a^ | 26.60±0.3^a^ |
| Taste (40) | 32.20±0.3^c^ | 32.80±0.2^c^ | 33.60±0.2^b^ | 34.00±0.2^b^ | 37.80±0.3^a^ |
| Odor (30) | 23.80±0.3^b^ | 23.80±0.2^b^ | 24.40±0.2^b^ | 24.80±0.2^b^ | 26.20±0.2^a^ |
| Total | 80.80±0.6^c^ | 81.40±0.5^d^ | 83.20±0.5^c^ | 84.80±0.4^b^ | 90.60±0.5^a^ |

**Table 5.** Orthogonal test results obtained for the optimization of the fermentation conditions.

| **Treatment Number** | **A.Fermentation Temperature/°C** | **B.Fermentation Time/h** | **C.Koji Inoculation Amount/%** | **D.Addition Amount of *Eucommia ulmoides* Leaf Superfine Powder/%** | **Sensory Score/Points** |
| --- | --- | --- | --- | --- | --- |
| 1 | 1 (32) | 1 (32) | 1 (0.6) | 1 (1.5) | 89.29 |
| 2 | 1 (32) | 2 (36) | 2 (0.8) | 2 (2.0) | 91.71 |
| 3 | 1 (32) | 3 (40) | 3 (1.0) | 3 (2.5) | 91.86 |
| 4 | 2 (34) | 1 (32) | 3 (1.0) | 2 (2.0) | 90.29 |
| 5 | 2 (34) | 2 (36) | 1 (0.6) | 3 (2.5) | 89.29 |
| 6 | 2 (34) | 3 (40) | 2 (0.8) | 1 (1.5) | 87.86 |
| 7 | 3 (36) | 1 (32) | 2 (0.8) | 3 (2.5) | 82.00 |
| 8 | 3 (36) | 2 (36) | 3 (1.0) | 1 (1.5) | 84.00 |
| 9 | 3 (36) | 3 (40) | 1 (0.6) | 2 (2.0) | 82.71 |
| K1 | 272.86 | 261.58 | 261.29 | 261.15 |  |
| K2 | 267.44 | 265.00 | 261.57 | 264.71 |  |
| K3 | 248.71 | 262.43 | 266.15 | 263.15 |  |
| k1 | 90.95 | 87.19 | 87.10 | 87.05 |  |
| k2 | 89.15 | 88.33 | 87.19 | 88.24 |  |
| k3 | 82.90 | 87.48 | 88.72 | 87.72 |  |
| R^2^ | 8.05 | 1.14 | 1.53 | 0.52 |  |
| Factor priority | A > C > B > D | | | |  |
| Optimal parameter combination | A_1_B_2_C_3_D_2_ | | | | |

**Table 6**. Result of optimization verification test for fermentation conditions of the new product.

| **Evaluation item** | **Ⅰ** | **Ⅱ** |
| --- | --- | --- |
| Color and shape (30) | 26.71 | 25.57 |
| Taste (40) | 35.29 | 36.14 |
| Odor (30) | 26.14 | 24.57 |
| Total | 88.14 | 86.29 |

Note: Ⅰ represents the orthogonal optimal combination; Ⅱ represents the single-factor optimal combination.

**Table 7.** Test results for the physical and chemical indexes and hygienic indexes of the new product.

| **Number** | **Testing Item** | **Unit of Measurement** | **Standard Requirement** | **Testing Result** | **Result** |  |
| --- | --- | --- | --- | --- | --- | --- |
| 1 | Alcohol content (20 °C) | %vol | - | 0.00 | qualified |  |
| 2 | Total sugar (calculated as glucose) | g/100g | - | 21.50 | qualified |  |
| 3 | Total acid (calculated as lactic acid) | g/kg | - | 6.10 | qualified |  |
| 4 | pH value | - | - | 3.92 | qualified |  |
| 5 | Lead (calculated as Pb) | mg/kg | ≤0.2 | 0.12 | qualified |  |
| 6 | Inorganic arsenic (calculated as As) | mg/kg | ≤0.2 | 0.052 | qualified |  |
| 7 | Aflatoxin B1 | μg/kg | ≤0.1 | Not detected | qualified |  |
| 8 | Total number of colonies | CFU/g | n = 5, c = 2, m = 10^2^, M = 10^4^ | <100 | qualified | |
| 9 | *Salmonella* | - | n = 5, c = 0, m = 0/25 | Not detected | qualified |  |
| 10 | *Staphylococcus aureus* | CFU/g | n = 5, c = 1, m = 10^2^, M = 10^3^ | <10 | qualified | |
| 11 | *Escherichia coli* | CFU/g | n = 5, c = 2, m = 10, M = 10^2^ | <10 | qualified | |
